# Connecting N6‐methyladenosine modification to ferroptosis resistance in hepatoblastoma

**DOI:** 10.1002/ctm2.820

**Published:** 2022-04-12

**Authors:** Wan‐Xin Peng, Yin‐Yuan Mo

**Affiliations:** ^1^ Pharmacology and Toxicology Cancer Institute University of Mississippi Medical Center Jackson Mississippi USA; ^2^ Department of Surgical Oncology National Clinical Research Center for Child Health National Children's Regional Medical Center Children's Hospital Zhejiang University School of Medicine Hangzhou China

1

Although hepatoblastoma (HB) rarely occurs in adults, it is the most common paediatric malignant liver cancer.^1^ Prognosis of HB is generally favourable; however, challenges remain for those unresectable tumours and/or chemotherapy resistant tumours. The underlying mechanism is not fully understood yet. In this issue, Liu et al. demonstrate that solute carrier family 7 member 11 (SLC7A11), a key component of system Xc‐ cystine/glutamate antiporter involved in ferroptosis,^2^ is subject to N6‐methyladenosine (m6A) modification. Furthermore, this m6A‐mediated regulation of SLC7A11 may contribute to HB progression and ferroptosis resistance (You need to add Liu at al. paper here. We did not list because we did not know when their paper will be published and in which issue).

It is well known that mRNA is subject to a variety of modifications and the most abundant internal RNA modification is m6A.^3^ Increasing evidence further indicates that m6A modification plays an important role in gene expression through RNA metabolism such as alternative splicing, mRNA stability, mRNA nuclear export, microRNA binding and RNA–protein interaction.^4^ The m6A modification is a dynamic process that involves three key players, that is, writers, erasers and readers.^3^ Of particular interest, these players have been implicated in cancer development and therapy resistance. Therefore, a better understanding of how m6A regulates expression of genes involved in a particular cellular pathway such as ferroptosis is of great interest.

Ferroptosis is a new type of cell death, different from apoptosis or necroptosis. A major player in ferroptosis signalling is the system Xc‐ that consists of light (SLC7A11) and heavy (SLC3A2) chain subunits. SLC7A11 exerts the main biological function and transport activity, whereas SLC3A2 serves as a chaperone protein to maintain the stability of SLC7A11. Specifically, SLC7A11 imports cystine from extracellular environment into the cell while exporting glutamate out of the cell simultaneously.^2^ After importing into the cell, cystine is reduced to cysteine to synthesize reduced glutathione (GSH) that is subsequently converted to oxidized glutathione (GSSG) by glutathione peroxidase 4 (GPX4). As a result, the cells become resistant to lipid peroxidation and ferroptosis. Therefore, SLC7A11 plays a critical role in ferroptosis resistance.

Since cancer cells are often under metabolic stress, an inducing factor for ferroptosis, this system is particularly important for cancer cells. To overcome this challenge, cancer cells have evolved various mechanisms to maintain ROS homeostasis through long periods of evolution against the host (human). Upregulation of SLC7A11 is one of such examples. Thus, the cancer cells would be able to survive in the harsh tumour microenvironment because GSH generated from the imported cysteine through SLC7A11 can neutralize excessive free radicals. In support of this notion, SLC7A11 is often overexpressed in a variety of tumour cells including HB. Now, an intriguing question is how SLC7A11 is regulated in cancer. Evidently, the regulation of SLC7A11 is complex, involving transcriptional, posttranscriptional and posttranslational regulations as well as epigenetic regulation (Figure 1). The study by Liu et al. focuses on epigenetic regulation of SLC7A11; specifically, they are interested in whether and how m6A impacts SLC7A11 expression.

**FIGURE 1:**
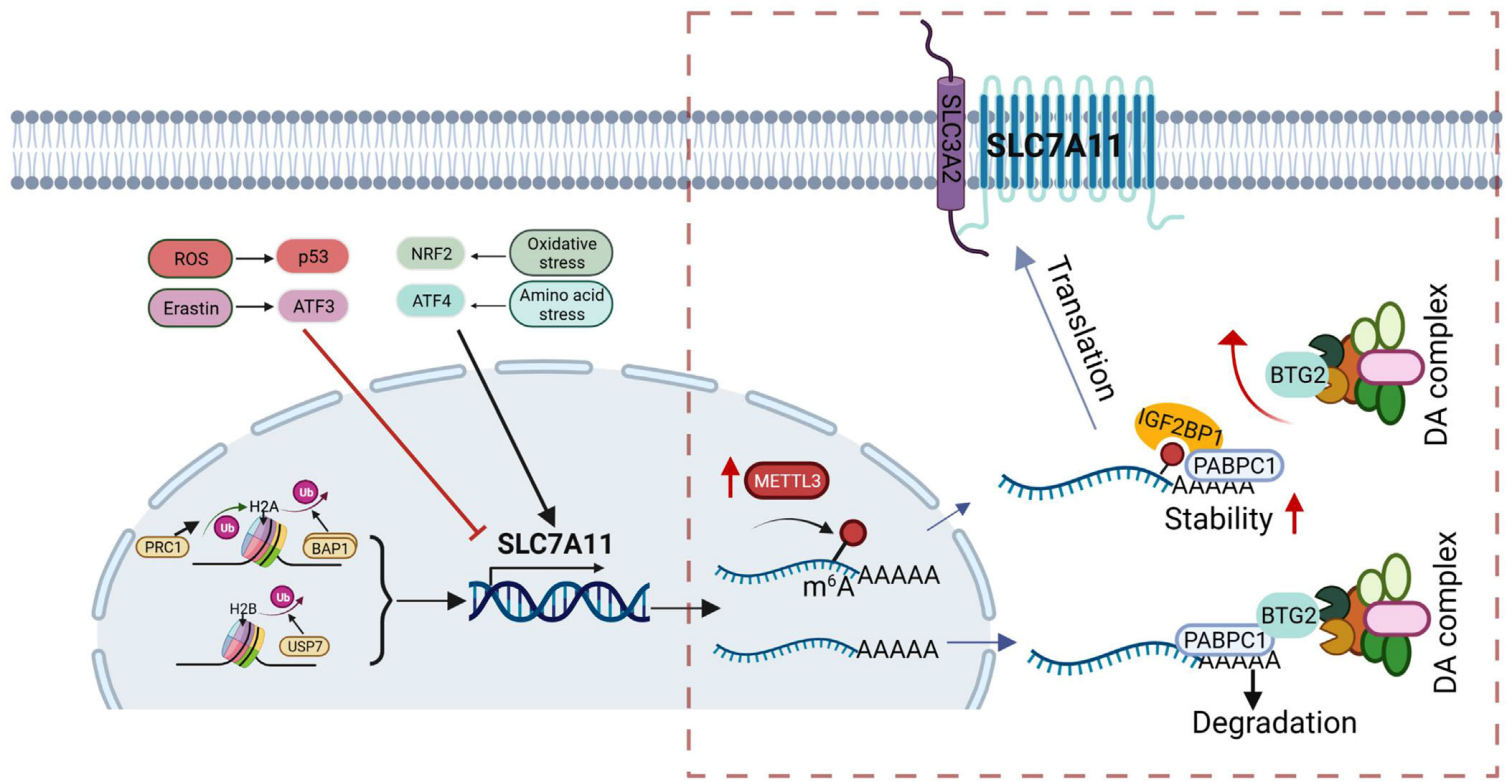
Regulation of SLC7A11. The finding by Liu et al. is highlighted in box. (1) Upregulation of METTL3 in HB leads to an increased level of m6A modification on SLC7A11. (2) Recognition of m6A‐modified SLC7A11 by IGF2BP1. (3) As a result, SLC7A11 is stabilized as DA complex is no longer able to interact with SLC7A11. DA complex, deadenylation complex involved in degradation of mRNA

First, RNA‐seq and MeRIP‐seq analyses reveal that SLC7A11 is one of the top overlapping genes. Next, functional study indicates that SLC7A11 siRNAs suppress tumour cell growth, and enhance cell death and lipid ROS production, which can be rescued by the ferroptosis inhibitor. Subsequently, they demonstrate that SLC7A11 is subject to m6A modification, which in turn regulates its expression. The abundance and effects of m6A on RNA are primarily determined by the dynamic interplay among writers, erasers and readers. However, this interplay is often dysregulated in cancer. Writers are methyltransferases that directly add methyl group to N6 position of adenosine. They usually have specific consensus motifs for RNA substrates. Three lines of evidence from their study suggest that METTL3 serves as a writer for SLC7A11 m6A modification in HB. (1) There is a positive correlation between METTL3 and SLC7A11. (2) The m6A modification of SLC7A11 mRNA was upregulated. (3) METTL3 KD reduces the SLC7A11 level, and enhances lipid ROS and the sensitivity of HB cells to ferroptosis.

A question is how m6A modification regulates SLC7A11. Remember that another important player of this m6A machinery is m6A readers. Readers recognize the m6A‐modified RNA molecules and their interaction with m6‐modified RNA molecules can lead to different functional consequences in a context‐dependent manner. Several members of specific RNA‐binding proteins have been shown to play such a role. Notably, YT521‐B homology (YTH) domain family proteins^5^ and IGF2 mRNA‐binding proteins (IGF2BPs)^6^ are major readers. By testing YTHDF2, YTHDC3 and IGF2BP1/2/3, they find that only IGF2BP1 is able to recognize mA6‐modified SLC7A11.

Now how does the recognition by IGF2BP1 lead to an increased level of SLC7A11? It turns out that like other mRNAs, SLC7A11 is also subject to mRNA decay mechanism, which is regulated by several factors, especially deadenylases. Among three deadenylation complexes tested, CCR4–NOT complex appears to play a key role in controlling SLC7A11 stability. At a low level of m6A modification, RNA‐binding protein PABPC1 at the poly(A) tail normally is capable of interacting with B cell translocation gene 2 (BTG2). This interaction would enable BTG2 to promote mRNA decay by recruiting CAF1 deadenylase,^7^ a subunit of CCR4–NOT complex. Although YTHDF2 has also been reported to destabilize m6A‐modified mRNAs,^8^ it does not seem to play a role in degradation of m6A‐modified SLC7A11 mRNA. Thus, through competitive interaction with PABPC1, IGF2BP1 blocks the recruitment of BTG2/CCR4–NOT complex by PABPC1, thereby suppressing the deadenylation of SLC7A11 mRNA and increasing its stability (Figure 1).

In summary, this study provides solid evidence that RNA methylation plays an important role in HB ferroptosis through regulation of METL3/SLC7A11/IGF2BP1 axis and thus, it may serve as a knowledge base to explore the clinical significance and therapeutic potentials. For example, can SLC7A11 and its m6A modification pathway be used for HB diagnosis/prognosis or a therapeutic target to treat HB patients who cannot benefit from current therapies? Given that β‐catenin is also subject to m6A modification in HB,^9^ it would be interesting to explore whether suppression of Wnt/β‐catenin signalling along with agents targeting the system Xc‐ or downstream signalling molecules would have synergistic effect on HB.

## CONFLICT OF INTEREST

The authors declare no conflict of interest.
